# Exploring urban growth–climate change–flood risk nexus in fast growing cities

**DOI:** 10.1038/s41598-022-16475-x

**Published:** 2022-07-18

**Authors:** Salah Basem Ajjur, Sami G. Al-Ghamdi

**Affiliations:** Present Address: Division of Sustainable Development, College of Science and Engineering, Hamad Bin Khalifa University, Qatar Foundation, Doha, Qatar

**Keywords:** Climate sciences, Environmental sciences, Hydrology

## Abstract

This study looks at the nexus between urban growth, climate change, and flood risk in Doha, Qatar, a hot-spot, climate change region that has experienced unprecedented urban growth during the last four decades. To this end, this study overviews the main stages of Doha’s urban growth and influencing climatic factors during this period. A physically-based hydrological model was then built to simulate surface runoff and quantify flood risk. Finally, the Pearson correlation was used to verify the potential nexus between flood risk, climate change, and urban growth. Surveying showed that, between 1984 and 2020, urban areas grew by 777%, and bare lands decreased by 54.7%. In addition, Doha witnessed various climatic changes with a notable increase in air temperature (+ 8.7%), a decrease in surface wind speed (− 19.5%), and a decrease in potential evapotranspiration losses (− 33.5%). Growth in urban areas and the perturbation of climatic parameters caused runoff to increase by 422%, suggesting that urban growth contributed more than climatic parameters. Pearson correlation coefficient between flood risk and urban growth was strong (0.83) and significant at p < 0.05. Flood risk has a strong significant positive (negative) correlation with air temperature (wind speed) and a moderate positive (negative) correlation with precipitation (potential evapotranspiration). These results pave the way to integrate flood risk reduction measures in local urban development and climate change adaptation plans.

## Introduction

Population increase, centralization, and industrialization typically push cities to urban growth. This growth can lead to severe environmental, social, and ecological consequences. Among environmental consequences, floods continue to be a big concern^1,2^. Urban growth implies a reduction of vegetation and bare lands coverage, an expansion of impervious surfaces, and a reduction in pervious surfaces. Such circumstances hinder groundwater recharge^3^, perturb evapotranspiration losses^4^, and accumulate surface runoff, raising the risk of floods^3,5^. Climate change, particularly precipitation extremes, is another factor that exposes populations and infrastructure to urban flooding, with a significant impact on poor people, children, and critical infrastructure^6,7^. Therefore, it is necessary to continually look at the nexus between urban growth, climate change, and flood risk. This necessity is of strategic importance in arid countries, which are generally characterized by frequent and intense rainfall^8^. At the same time, they either have no or poor stormwater drainage systems.

A growing body of literature has documented the relationship between urban growth and flood risk. Di Baldassarre, et al.^9^ linked flood damage with intensive and unplanned urban development in Africa. They recommended discouraging people from settling in flood-prone areas and highlighted the need for early warning systems. Lee and Brody^10^ highlighted the role of haphazard urban development in causing floods in Seoul, South Korea. They recommended planning resilient urban development to help alleviate flood losses. As a result of urban growth in the southern river catchment of Western Australia, the runoff coefficient has increased from 0.01 to 0.4, significantly raising flood risk in the area^11^. The relationship between climate change and flood risk has also gained researchers’ attention. Ahmed, et al.^6^ found that climate change has a more significant impact on flooding than unplanned urban growth in Dhaka, Bangladesh. In contrast, Sofia, et al.^12^ demonstrated that though climate change impacts flood risk, land use in northeastern Italy still has a significant impact. While one cannot put too much weight on the impact of urban growth or climate change on flood risk, it can be concluded that additional efforts must be devoted to expanding current knowledge about this nexus. Specifically, efforts need to be made to show how flood risk shifts in the context of urban growth and climate change impacts.

This study investigates the nexus between urban growth, climate change, and flood risk in Qatar during the last four decades to address this knowledge gap. Qatar offers an excellent opportunity to explore such a nexus for several reasons. Qatar’s population has increased rapidly since the discovery of oil and gas in the 1970s. The main population-associated problems are rapid urbanization, pollution increase, and depletion of natural resources. The largest city in Qatar, Metropolitan Doha, has radically transformed from small fishing and pearling settlements to a highly urbanized, dominant city to improve living standards and be ready to host the 2006 Asia Games and the 2022 FIFA World Cup. However, the environmental implications of Doha’s urban growth have not been discovered. Rizzo^13^ approximated that the built environment in Doha grew 60 times in only half a century, a growth that may have outpaced any other region on the planet. Rizzo^14^ argued the country’s inability to manage the consequences of such urban growth. Shandas, et al.^15^ researched urban growth patterns in Doha and recommended investigating the negative implications of such growth for humans and the environment. In addition, Qatar is a climate change hot spot (see “Case study description”) making it a good example to explore urban growth, climate change, and flood risk nexus.

Accordingly, this study revisits the nexus between urban growth, climate change, and flood risk in Doha between 1984 and 2020. First, a physically-based hydrological model (WetSpass) was applied to investigate water balance components and quantify surface runoff. After that, the Pearson correlation was used to verify the potential nexus between urban growth, climate change, and flood risk. The study findings are vital to supporting flood risk reduction policies by integrating them into local urban development and climate change adaptation plans. Furthermore, the proposed approach can be applied to similar climatic regions characterized by rapid urban development.

### Case study description

Qatar is located between 24° 16′–26° 6′ North and 50° 27′–51° 24′ East. It covers 11,651 km^2^ with an increasing elevation from zero at coasts to up to 92 m on the southeast side. Historically, Qatar has had a relatively small population, but the population has rapidly grown from 0.22 million in 1980 to 2.88 million in 2020^16^. Approximately 85% of the population lives in Doha, the capital city. Qatar's population is expected to increase to 3.85 million by 2050 under the medium-fertility variant scenario^16^.

Qatar is classified by a hyper-arid climate with intense minimal rainfall, scorching air temperature, and very high relative humidity^17^. Previous literature has projected that Qatar will experience heavier rain and an increase of up to 6 °C in average air temperature before 2100^18^. Mamoon and Rahman^19^ analyzed the spatiotemporal distribution of Qatar rainfalls between 1962 and 2010. They found that both rainy days and total winter rainfall are increasing. At the same time, Qatar has a poor stormwater drainage system, comprised of some subsurface chambers and pipes where excess runoff is diverted. These conditions led to flash floods during heavy storms in 1995, 2015, and 2018. Within only 24 h, Dukhan municipality recorded 123 mm of rain on 12 March 1995, Hamad Airport recorded 81 mm of rain on 25 November 2015, and Abu Hamour recorded 84 mm of rain on 20 October 2018^20^. Some of these floods caused fatalities and several injuries, not to mention catastrophic asset and property losses.

## Data

Analyzing US Geological Survey Landsat satellite images determined seven significant changes in Doha land use. These changes were documented at five-year increments that correspond to the years: 1990, 1995, 2000, 2005, 2010, 2015, and 2020. For each year, the corresponding images were obtained from the Landsat at a resolution of 30 m. We selected images acquired in June with a cloudiness ratio of less than 5%. Accordingly, we represented the main stages of urban growth in Doha by analyzing seven periods starting from 1984 and ending by 2020. We selected 1984 as a starting year because neither Landsat images nor Qatar meteorological station climatic records were available before 1984. The seven studied periods and their corresponding land use images, paths, and rows are tabulated in Table 1.

**Table 1:** Information on the seven periods and landsat images.

Periods	Time frame	Sensor name/Product name	WRS Path	WRS Row	Acquisition date	Number of bands	Cloud cover (%)
P1	1984–1990	Landsat-5-TM/L1TP	163	42 and 43	6 June 1990	6	0
P2	1991–1995	Landsat-5-TM/L1TP			4 June 1995	6	1
P3	1996–2000	Landsat-5-TM/L1TP			17 June 2000	6	1
P4	2001–2005	Landsat-7-ETM + /L1TP			7, 23 June 2005	6	5
P5	2006–2010	Landsat-7-ETM + /L1TP			21 June 2010	6	0
P6	2011–2015	Landsat 8/L1TP			11 June 2015	8	0
P7	2016–2020	Landsat 8/L1TP			8 June 2020	8	0

**Figure 1:**
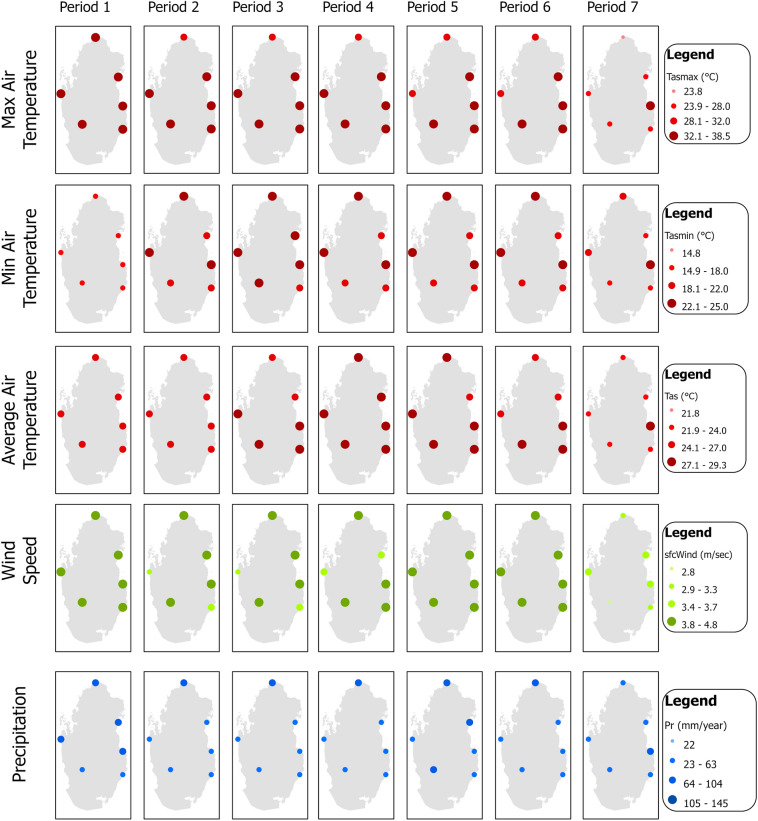
Mean annual climatic observations at Qatar weather stations.

The study also obtained five monthly climatic parameters during 1984 and 2020 from six meteorological stations managed by the Qatar Civil Aviation Authority. These parameters are maximum, minimum, and average air temperature; wind speed; and precipitation. Although no systematic changes in stations’ locations or measurement methods occurred during the last four decades, the Civil Aviation Authority has screened climatic records for accuracy and consistency to avoid data inhomogeneity. Figure 1 shows mean annual climatic observations at the weather stations averaged over each studied period in Table 1. For example, during Periods 1–7, Qatar maximum air temperature ranged between 296.9 (23.8 °C) and 311.6 K (38.5 °C) with an average of 305.8 K (32.7 °C), minimum air temperature ranged between 287.9 (14.8 °C) and 298.1 K (25 °C) with an average of 294.1 K (21 °C), and mean air temperature ranged between 294.9 (21.8 °C) and 302.4 K (29.3 °C) with an average of 299.7 K (26.6 °C). In addition, surface wind speed varied between 2.8 and 4.8 m/s with an average of 3.9 m/day, while annual rainfall records had higher spatial variability ranging between 22 and 145 mm with an average of 62.1 mm. In general, northern stations had higher rainfall than southern stations.

## Methodology

The research methodology for this study consists of four steps. First, land use maps representing the seven periods were prepared and pre-processed. The Landsat images were compiled and mosaicked-based satellite imagery interpreted using the supervised classification method. A very high accuracy (89%) was obtained from the supervised classification. The 2020 Landsat images were updated with a current land cover map obtained from the Ministry of Municipality and Environment (MME). The original land use categories were then reclassified into three main classes: urban areas, vegetation areas, and bare lands. Urban areas include built-up surfaces such as residential and commercial facilities, roads and utilities, construction sites, and industrial communities. Vegetation areas include farms, parks, golf courses, and trees. Bare lands are mainly lithosols composed of thin, calcareous, sandy loams (up to 30 cm) covered by limestone debris and bedrock^21^.

Second, average, maximum, and minimum air temperatures were used to compute monthly potential evapotranspiration (PET; see “Potential evapotranspiration (PET) estimation”). Monthly PET and climatic parameters were then averaged throughout each year to obtain seasonal trends (except rainfall records were aggregated). April–September represented the summer season, while October to March represented the winter season. Afterward, summer and winter data were averaged across years for each period mentioned in Table 1. For instance, in the first period, the average summer temperature is the mean value of April to September during 1984–1990, while the average winter temperature is the mean value of October to March during 1984–1990. Next, each station’s influence on the whole country was weighted using the inverse distance weighted interpolation^2,12^. As a result, we had mean summer and winter climatic maps for the seven periods. Since hydrological estimations can be affected by the spatial resolution of input maps^22^, all the maps were reclassified into a standard spatial resolution of 30 × 30 m.

The third step involved implementing a physically-based, spatially distributed, hydrological model (WetSpass) to calculate surface runoff on the country scale. WetSpass is a reliable model to study hydrological cycle components based on the water balance concept^3–5,22–24^. In WetSpass, precipitation measurements are partitioned between runoff, groundwater recharge, and actual evapotranspiration (AET). The model requires setting four climatic parameters (temperature, rainfall, wind speed, and PET), physical catchment properties (slope, soil type, and land use), and groundwater properties (groundwater level depth). The soil and Digital Elevation Model (DEM) maps were obtained from the MME, while groundwater level data was obtained from fieldwork done by Schlumberger Water Service in 2018. The slope map was derived from the DEM. The reader can find more details about WetSpass in Batelaan and De Smedt^24^.

Fourth, Pearson’s correlation was used as a way to verify the potential relationship between Doha urban growth, climate change, and flood risk^5,25,26^. Urban growth was represented by the percentage of urban areas in the seven studied periods, while the simulated runoff represented flood risk. Finally, climate change was characterized by climatic factors used in hydrological modeling. The above methodology was executed using a geographic information system (GIS).

### Potential evapotranspiration (PET) estimation

This study calculated PET using Hargreaves and Samani^27^ equation. Hargreaves and Samani^27^ equation is a temperature-based equation widely used to estimate PET for agriculture, water resource, and climate impact studies^28,29^. Though using a standard method, such as the Penman–Monteith equation, has maximum climatic variable coverage and may better estimate PET, the Penman–Monteith equation requires extensive inputs that are not fully available at weather stations. Further, several studies reported comparable, reliable results obtained using the Hargreaves and Samani^27^ equation to those obtained using the Penman–Monteith equation in various environments^27^. Therefore, this study used the Hargreaves and Samani^27^ equation to calculate the monthly PET as follows:

1 $$PET ={N}_{d}{\times 0.0023 R}_{a} \left(T+17.8\right) \sqrt{Tmax-Tmin}$$

where *T, T*_*max*_, and *T*_*min*_ represent mean, maximum, and minimum temperature in ^°^C, respectively, N_d_ represents the number of days in each specific month, and *R*_*a*_ represents the water equivalent of extraterrestrial radiation in mm/day. Extraterrestrial radiation is computed from the latitude and day data of the year^30^. The monthly PETs were then aggregated to get summer and winter PETs and used as inputs for WetSpass.

## Results

This section presents how Doha’s land use categories changed through the seven studied periods. The section then links flood risk development with climate and land-use changes and determines the urban growth-climate change-flood risk nexus.

### Land cover development between 1984 and 2020

**Figure 2:**
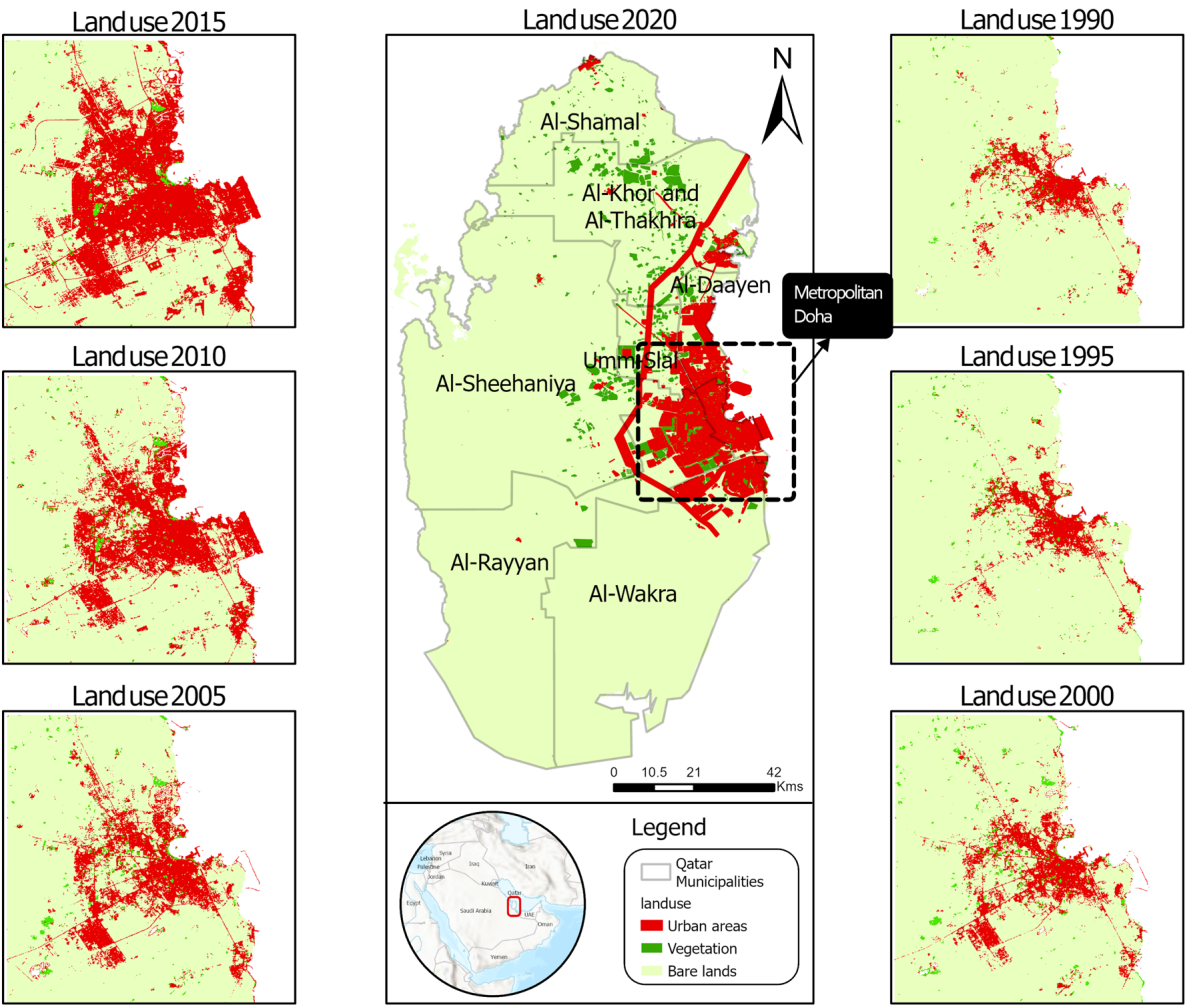
The general location of Qatar with observed expansion in Qatar urban development during the seven periods. The black box in the middle map shows metropolitan Doha. Maps were created using ArcGIS Pro version 2.9 (https://www.arcgis.com/).

Figure 2 depicts the spatial distribution of the three land-use patterns in the seven studied periods. Minimal urban growth was observed between Periods 1 and 2. Early adjacent urban growth of 39 km^2^ was observed during Periods 2 and 3. This growth was sporadically distributed over the area. Urban development continued after 2001 with an expansion of 36.1 km^2^ between 2001 and 2006, mainly in the central business district, the west bay area. After 2006, around 58 km^2^ of urban areas were developed around mega-projects such as the Pearl and Hamad International Airport. Last, infill development and sustainable growth opportunities led to the most remarkable expansion in urban and vegetation areas, which occurred in the outskirts of Doha during the last two periods. This growth makes Doha more agglomerated and less fragmented. The analysis showed that approximately 107 km^2^ and 126.6 km^2^ of urban areas were developed between 2011–2016 and 2016–2020.

**Figure 3:**
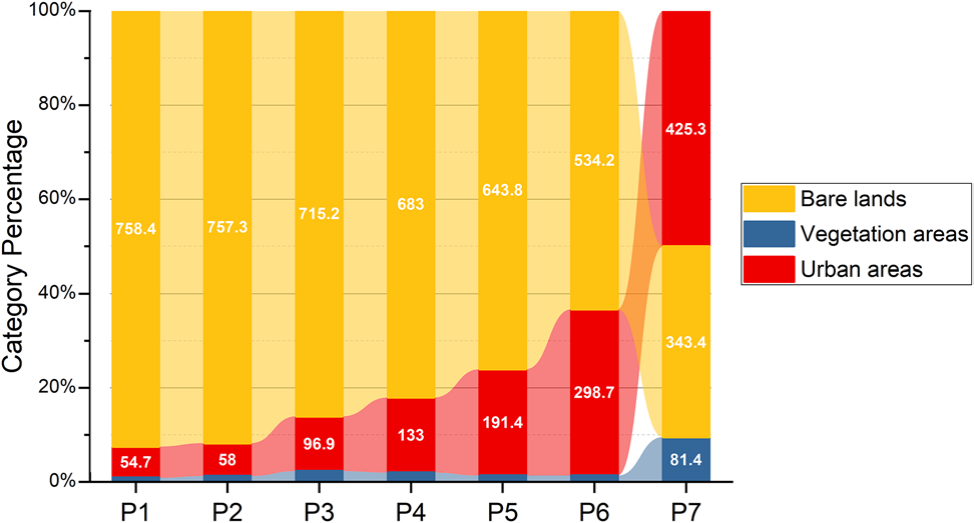
Changes between the three land-use classes in Doha during the seven studied periods. Labels show the area of each category in km^2^.

Figure 3 depicts changes in land cover classes from 1984 to 2020, as determined by analyzing the Landsat images. Generally, Doha witnessed a significant increase (from 54.7 to 425.3 km^2^) in urban areas, which amounts to 777% growth over the period. The most notable increase (15%) was found between 2016 and 2020 (Period 7) along the Salwa Highway and the Doha Expressway. Considerable coastline modification occurred at the Pearl and Hamad International Airport during Periods 4 and 5. In contrast, bare lands have decreased from 758.4 to 343.4 km^2^ since 1984, reaching a 54.7% decline over the period. In the first six periods (P1–P6), bare lands was the largest land-use class in Doha. However, bare lands decreased to 40%, and urban areas increased to 50% in the past five years (P7). The Doha area does not naturally contain vegetation. In 1984, the vegetation coverage was only 7.3 km^2^, covering only 1.3% of Doha. However, vegetation coverage increased, especially after 2016, to 81.4 km^2^ covering 9.6% of the Doha area. This statistic indicates the recent efforts of urban planners to expand parks and green landscape coverage in the last five years. Vegetation areas never exceeded 19.3 km^2^ before 2016. Accordingly, the proportion of vegetation areas to urban areas exhibited a minor increase (from 13.4 to 19.9%) during P1 and P3, while it decreased to 3.9% in Period 6. During the last period, the proportion of vegetation areas to urban areas had the highest record of 19.1%.

### Climate change between 1984 and 2020

**Figure 4:**
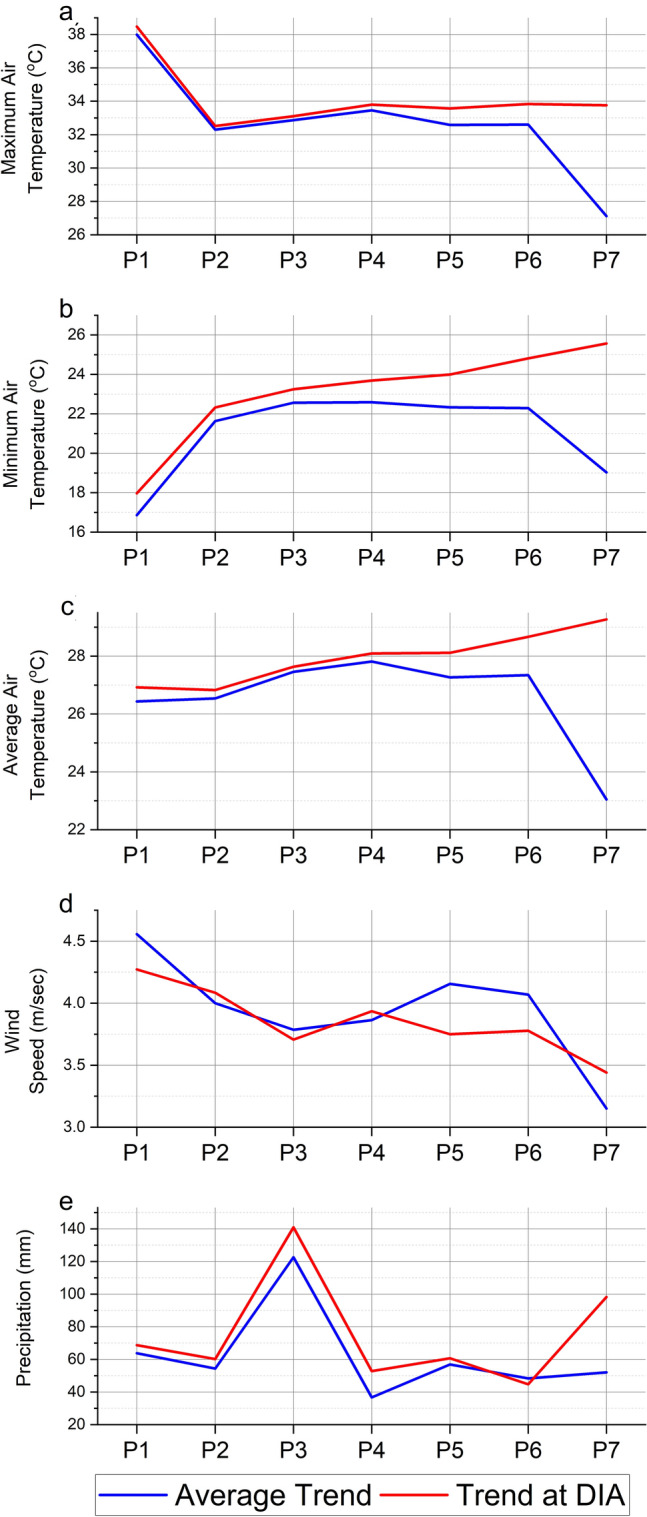
The trend in mean annual maximum, minimum, and average air temperatures, wind speed, and precipitation during the studied periods averaged over Qatar (blue lines) and at Doha International Airport station (red lines).

Figure 4 shows the annual climatic parameters averaged over Qatar and at the Doha International Airport (DIA) during the seven studied periods. Both trends (Qatar and DIA average) are similar; however, there are some variations, especially during the last period. These variations imply a variant rainfall distribution throughout the region^8^. In Qatar, maximum air temperatures varied, with 38 °C being the highest observed temperature in Period 1 and 27.1 °C being the lowest observed temperature in Period 7. The overall decrease in maximum air temperature was 10 °C. Qatar’s mean minimum air temperature varied slightly among the seven periods. It increased by 5.7 °C from Period 1 to Period 4 then decreased by 3.6 °C. Between Periods 1 and 4, average air temperature increased from 26.4 to 27.8 °C (1.4 °C) but then fell by 4.8 °C through Period 7. Wind speed values oscillated between 3.15 m/s in Period 7 and 4.56 m/sec in Period 1. Precipitation decreased slightly after Period 5. The highest amount of rain (122.6 mm/year) was recorded in Period 3, while the lowest amount (36.7 mm/year) was recorded in Period 4. The last two periods had minimal amounts of rainfall. On average, Qatar received 62.1 mm/year of rain between 1984 and 2020.

At the DIA, maximum air temperature decreased by 4.8 °C (from 38.5 to 33.7 °C), minimum air temperature increased by 7.6 °C (from 18 to 25.6 °C), and average air temperature increased by 2.4 °C (from 26.9 to 29.3 °C), through the whole period. Wind speed values oscillated between 3.4 m/s in Period 7 and 4.27 m/sec in Period 1, with an average of 3.85 m/s. The highest recorded yearly precipitation was found in Period 3, hitting 141 mm, whereas other periods (except Period 7) recorded lower annual precipitation that did not exceed 68.3 mm. On average, DIA received around 75.2 mm of annual rain during the seven periods, exceeding the country’s average by 13.1 mm.

### Hydrological analysis

**Figure 5:**
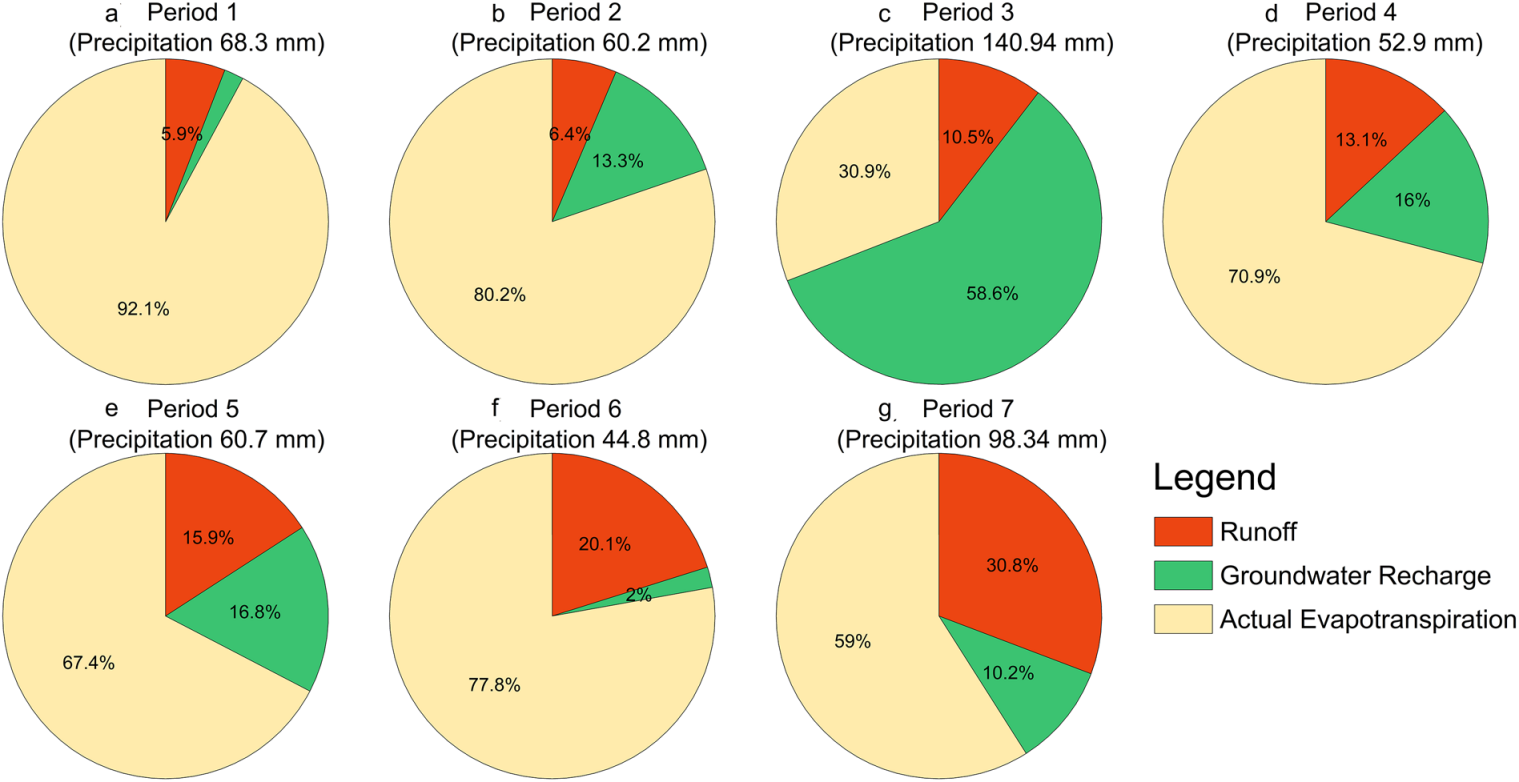
The distribution of Doha rainfall between runoff, groundwater recharge, and actual evapotranspiration during the seven studies periods. Annual rainfall is shown under each period name. The maps were created using Python 3.9 (https://www.python.org).

Figure 5 shows the distribution of Doha rainfall between runoff, groundwater recharge, and AET. In general, most of Doha’s rain is lost to AET due to the hyper-arid environment. However, the proportion of AET to rain decreased with time because runoff increased. While only 5.9% of rain flowed as runoff and AET constituted 92.1% of precipitation in Period 1, the proportion of AET decreased to 59% in Period 7. This analysis shows that urban growth influences groundwater recharge more than rainfall. In Period 3, rain hit a high value of 141 mm/year, generating 58.6% of groundwater recharge. On the other hand, Period 7 had high rainfall, 98.34 mm/year (compared to other periods), while its groundwater recharge did not exceed 10.2%. In other words, the latest urban growth in Period 7 had a significant impact on groundwater recharge by hindering remarkable amounts of rainfall from feeding the aquifers.

Generally, the “high-to-low” sequence of runoff is urban areas, vegetation areas, and bare lands, respectively. Therefore, the runoff process is highly responsive to urban development. If urban areas grow, the runoff will increase. In Doha, urban areas have grown since 1984, with significant growth during Period 6 and Period 7. Consequently, the proportion of runoff to rain improved from 5.9% in Period 1 to 20.1% in Period 6 and 30.8% in Period 7. Runoff increased ~ 25% during the whole studied period. A remarkable increase in runoff can be noticed during the last two periods. For example, runoff increased by 4.2% between Period 5 and Period 6 and 10.7% between Period 6 and Period 7. These statistics reflect a significant increase in the runoff because of urban growth development.

Most of the annual runoff calculations were generated during the winter when most of the rain fell. During the summer, some areas had negative groundwater recharge, which implies that the summation of runoff and AET is higher than the precipitation. This lack of groundwater recharge occurs in zones with a shallow water table (near the land surface), where plant roots can penetrate the saturated zone and transpire water directly from aquifers. Overall, the generated quantities of runoff reached 0.28 million cubic meters (Mm^3^) and 0.25 Mm^3^ in Period 1 and Period 2, respectively. These quantities developed to 0.92 Mm^3^ in Period 3, 0.53 Mm^3^ in Period 4, and 0.65 Mm^3^ in Period 6. The highest runoff (1.75 Mm^3^) was found in Period 7.

**Figure 6:**
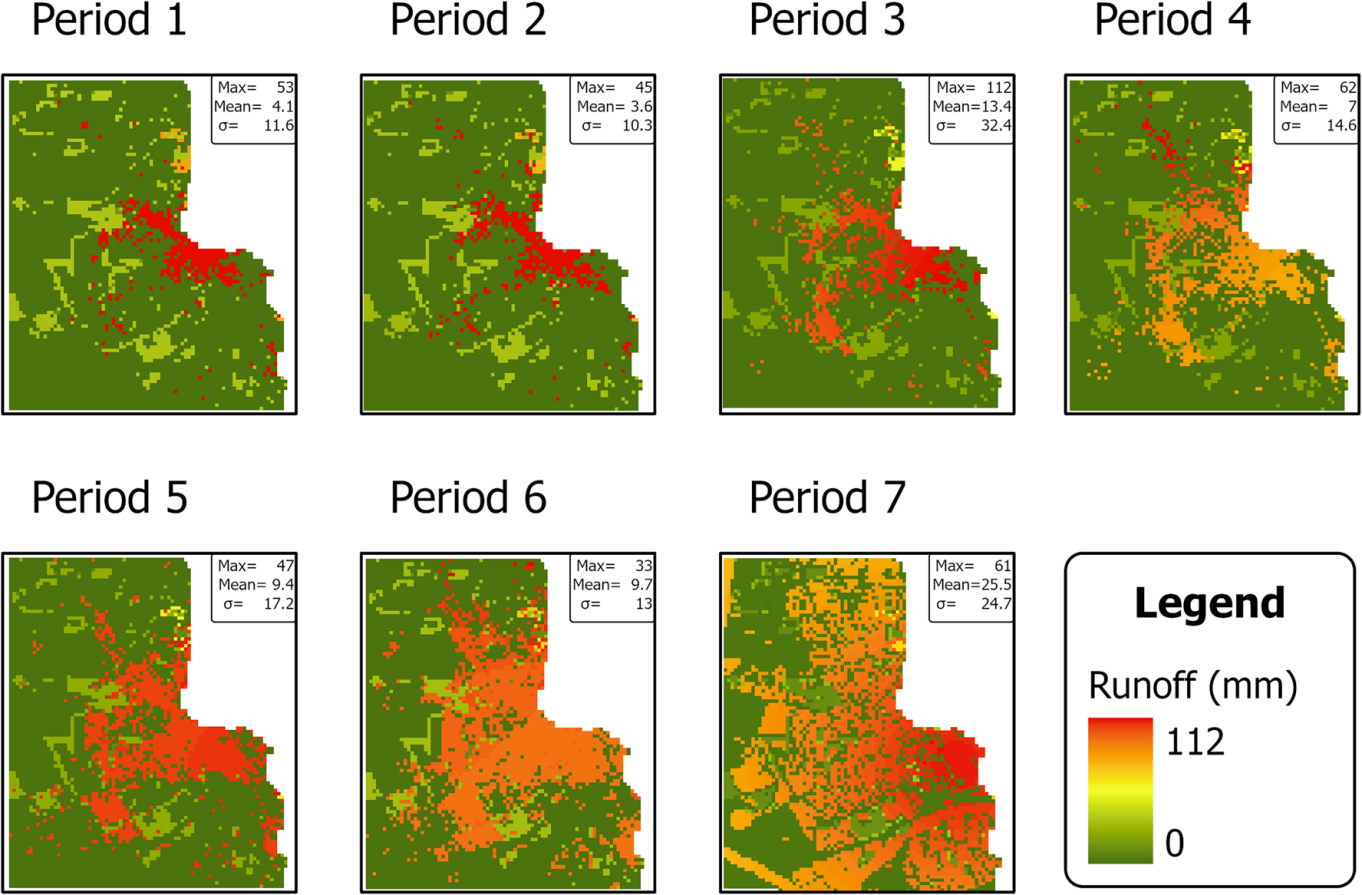
The spatial patterns of average annual surface runoff for the seven studied periods. Boxes in the right top corners show maximum (max), mean (Mean), and standard deviation (σ) values. Maps were created with ArcGIS Pro version 2.9 (https://www.arcgis.com/).

Figure 6 depicts average annual runoff spatial patterns in the seven studied periods. There are apparent spatial variations in runoff following land cover distribution. Higher runoff is correlated with the development of urban areas, implying a positive relationship between urban growth and runoff percentages. Apart from Period 3, maximum annual runoff varied up to 62 mm in all periods. Mean annual runoff did not exceed 9.7 mm in Periods 1, 2, and 4–6. Meanwhile, records of mean annual runoff hit 13.4 mm in Period 3 and 25.5 mm in Period 7. The mean annual runoff in Period 3 can be attributed to the high rainfall across the area (see Fig. 4). The mean annual runoff in Period 7 was the greatest among all the periods, indicating urban growth’s impact on generating runoff.

### Urban growth–climate change–flood risk nexus

This study used the Pearson’s correlation coefficients to measure the strength of the relationship between urban growth, climate change, and flood risk. Since the past growth patterns in Doha were primarily urban, urban growth was represented by the percentage of urban areas in the seven studied periods. The simulated amount of runoff represented flood risk in each period. In the seven periods, precipitation has a standard deviation of 33.54 mm, average air temperature has a standard deviation of 0.88 °C, wind speed has a standard deviation of 0.27 m/s, PET has a standard deviation of 8.27 mm/year, urban areas percentage has a standard deviation of 16%, and simulated runoff has a standard deviation of 0.512 Mm^3^/year. Table 2 describes the correlation coefficients between urban growth, climate change, and flood risk parameters. The correlation coefficient between flood risk and urban growth was 0.83 (significant at p < 0.05), implying that the increase in urban areas will amplify flood risk. Various climate change parameters correlated differently with flood risk in Doha. The flood risk correlation with precipitation was moderate and positive (non-significant) and was moderate and negative with PET. Flood risk had a strong positive correlation with surface air temperature and a strong negative correlation with surface wind speed. Both correlations (air temperature and wind speed) were significant at p < 0.05.

**Table 2:** The correlations flood risk has with urban growth and climate change. Sample size (n) = 7.

Correlation with flood risk	Climatic parameters				Urban growth
	Precipitation	Average air temperature	Wind speed	Potential evapotranspiration	
Correlation coefficient	0.52	0.81	− 0.90	− 0.49	0.83
p-value	0.23	0.027	0.0054	0.27	0.02

## Discussion

This study focuses on how urban growth and climate change impact flood risk in Doha. Previous literature documented similar variations among water balance components (e.g., groundwater recharge, surface runoff, and evapotranspiration) due to urban growth and climate change. For instance, Zomlot, et al.^5^ concluded that surface runoff is strongly affected by land use and soil types. Zhang, et al.^3^ concluded that land-use changes could significantly affect the spatial distribution of surface runoff in Beijing, China. Eini, et al.^31^ documented that urban texture had a significant influence on flood risk. Weatherl, et al.^32^ reported that runoff increase can be accompanied by AET decrease in urban areas. The positive correlation between rainfall and groundwater recharge is a good general rule^5^.

Our analysis demonstrated that the recent growth pattern in Doha was predominantly urban; urban areas grew 7.7 times between 1984 and 2020, from 54.7 to 425.3 km^2^. These results align with Hashem and Balakrishnan^33^, who, between 1997 and 2010, reported a 289% increase in built-up areas in Doha. In addition, our analysis indicates an increase of 230% in Doha urban areas between 1995 and 2010. However, study results disagree with Rizzo^13^, who estimated that, between the 1970s and 2013, the built-up areas in Doha increased by 60 times^13^.

The projected future increase in the Qatar population suggests enhanced urban growth demand. However, results obtained from developing high-resolution spatially probabilistic forecasts of urban growth are inconsistent. For example, Hashem and Balakrishnan^33^ projected future changes in Doha land use between 2010 and 2020 and expected a 55% reduction of farms and an 11% reduction of recreational areas. However, the current cadastral land use map reveals that vegetation areas increased by 8.3% between 2010 and 2020. In addition, this study could not obtain a clear vision of future urban development from urban planners or projected population increases, introducing significant uncertainty in runoff estimations^22^. Therefore, this study did not cover the future impact of urban growth. Similarly, as part of the Arabian Gulf, Qatar is projected to have uncertain climatic changes, especially rainfall projections^8^. Uncertainty in climate change could introduce uncertainty in flood risk assessment. Combined with future land growth uncertainty, a mismatch with actual changes and plan failures are expected when studying future relationship between urban growth, climate change, and flood risk^34^.

The projected increase in flood risk is particularly alarming for urban hubs in the Arabian Gulf. Though the Arabian Gulf is a unique and fragile system, urban hubs are growing several times faster than the international average^16,35^, with decision-makers wanting to achieve higher levels of development and prosperity. Therefore, the urban growth-climate change-flood risk nexus in these urban hubs must be managed. Also, since most urban development occurs along coasts in the Arabian Gulf, climate change puts these hubs at additional risk of rising sea levels^1,36^. Hence, unless wise and proactive flood and sea-level rise mitigation and adaptation actions are taken, the environmental cost of urban growth and climate change will exacerbate, and tipping points may be reached in the region’s physical systems.

## Conclusion

This study provides a sound understanding of the role of urban growth and climate change in increasing the flood risk in Doha. While urban growth is essential to meeting the population’s needs, study findings highlight the importance of shifting towards a more resilient integrated urban design based on evading the impact of climate change and alleviating flood risk. An integrated urban design must increase spaces that limit floods and enable more precipitation to recharge aquifers. Implementing land use planning and zoning strategies that include physical flood protection at regional and local scales can also go a long way in achieving sustainable flood risk reduction. Last, efforts should be devoted to combating climate change and its impact. Further analysis is required to reveal how fast urban growth activities affect public health, social life, and marine ecosystems. While Qatar is a case study, this paper can be an example for other countries with similar climate and environmental conditions.
